# Prevalence of intestinal parasitic contamination in fresh vegetables in Bangkok, Thailand, and surrounding areas: A cross-sectional survey

**DOI:** 10.1016/j.parepi.2025.e00416

**Published:** 2025-01-30

**Authors:** Pokkamol Laoraksawong, Uthaitip Bunkasem, Anunya Pradidthaprecha

**Affiliations:** a Department of Occupational Safety and Environmental Health, Faculty of Public Health, Khon Kaen University, Khon Kaen, Thailand; b Department of Parasitology, Faculty of Medicine, Chulalongkorn University, Bangkok, Thailand; c School of Health Science, Sukhothai Thammathirat Open University, Nonthaburi, Thailand

**Keywords:** Intestinal parasites, Fresh vegetable contamination, Thailand, Market hygiene practices

## Abstract

Intestinal parasitic infections are a major public health issue worldwide, and vegetables contaminated with these parasites have been implicated in their transmission. This study investigated the prevalence and distribution of intestinal parasites (IPs) on fresh vegetables in Bangkok, Thailand, and surrounding areas. This cross-sectional survey was conducted from November 1 to December 31, 2022. Vegetable samples were purchased from 12 markets in Bangkok and on surrounding areas. A total of 1800 fresh vegetable samples were collected and examined using direct wet-mount microscopy by parasitologists. The total prevalence of IPs in fresh vegetables was 21.17 %, with the highest prevalence observed in peppermint (79.17 %), followed by Thai basil (72.50 %) and *Centella asiatica* (40.83 %). Soil-transmitted helminths were predominantly detected, including hookworm larvae (13.06 %), *Strongyloides* spp. (rhabditiform larvae) (6.61 %), and *Ascaris lumbricoides* (2.50 %). Furthermore, open-air markets in rural areas had a 1.40 times higher chance (95 % CI: 1.10–1.74, *P* = 0.005) of IP contamination in vegetables than structured markets in urban areas. Additionally, fresh products in rural open-air markets were 8.54 times more likely to be contaminated with *Blastocystis* sp. (95 % CI: 2.57–28.40, *P* < 0.001) and 2.46 times more likely to be contaminated with Taeniidae spp. (95 % CI: 1.01–5.95, *P* = 0.036) compared to produce from structured markets in urban areas. The presence of these parasites in fresh vegetables highlights the need for improved food safety measures, including proper hygiene practices during vegetable cultivation, harvesting, transportation, and storage. Public health education campaigns on the risks of consuming contaminated vegetables should also be implemented to reduce the burden of intestinal parasitic infections in Thailand.

## 1 Introduction

Intestinal parasitic infections, particularly soil-transmitted helminths, remain a major public health problem worldwide, especially in lower- and middle-income countries (Duedu et al., 2014; World Health Organization, 2002). These infections can cause diarrhea, malnutrition, anemia, and even death, particularly among vulnerable populations, including young children, pregnant women, the elderly, and immunosuppressed individuals (Brooker et al., 2004; Djuardi et al., 2021; Schär et al., 2013). Ingestion of contaminated food and water is a common transmission route for intestinal parasites (IPs), including protozoa and helminths (Yahia et al., 2023). Vegetables are a known source of contamination with these parasites, as they are often consumed raw or minimally processed (Altwaim et al., 2023; Bekele and Shumbej, 2019; Kpoda et al., 2022; Punsawad et al., 2019). Several studies have documented the presence of IPs in fresh vegetables across various regions, including Asia, Africa, Europe, and South America (Abougrain et al., 2010; Bekele and Shumbej, 2019; Caradonna et al., 2017; Falcone et al., 2023; Ferreira et al., 2018; Gemechu et al., 2023; Robertson and Gjerde, 2001; Sia Su et al., 2012; Yahia et al., 2023). Both intestinal protozoa and helminths, such as *Blastocystis* sp., *Entamoeba* spp., *Giardia* spp., *Ascaris* spp., *Taenia* spp., hookworm larvae, *Strongyloides* spp., and *Trichuris* spp., have been reported in contaminated fresh vegetables (Abougrain et al., 2010; Falcone et al., 2023; Mohamed et al., 2016; Punsawad et al., 2019). Notably, the highest prevalence of protozoan contamination has been observed in Asia (Eslahi et al., 2024).

Thailand, classified as a middle-income country, faces a persistent challenge with parasitic infectious diseases in humans. Previous studies have reported the prevalence of human IP infection in Thailand as ranging from 9.8 % to 37.2 %, with soil-transmitted helminths and protozoa being particularly common (Assavapongpaiboon et al., 2018; Boonjaraspinyo et al., 2022; Boonjaraspinyo et al., 2013; Laoraksawong et al., 2018; Wattanawong et al., 2021). Fresh vegetables have been identified as a source of contamination with parasites such as *Blastocystis* sp., *Entamoeba* spp., hookworm larvaes, *Strongyloides* spp., *A. lumbricoides*, and *Taenia* spp. (Falcone et al., 2023; Obebe et al., 2020; Alemu et al., 2020; Mirzaei et al., 2021; Zeynudin et al., 2024), posing a risk of infection to humans who consume them raw, inadequately washed, or improperly prepared (Bekele et al., 2017). In Thailand, studies have reported intestinal parasitic contamination in fresh vegetables across various regions. Notable findings include prevalence rates of 50.8 % in the central region, such as Phra Nakhon Si Ayutthaya (Jongkolnee and Songthamwat, 2015), and 36.0 % in Nakhon Nayok (Krainara et al., 2023). Rates were also reported as 45.0 % in the northern region (Jinatham et al., 2023), 36.0 % in the northeastern region (Poochada et al., 2024), and 35.1 % in the southern region (Punsawad et al., 2019). In rural areas, fresh vegetables from open-air markets showed contamination rates between 35.1 % and 66.7 % (Niamnuy et al., 2022; Poochada et al., 2024; Punsawad et al., 2019), whereas structured markets in urban areas exhibited contamination rates ranging from 25.0 % to 45.0 % (Niamnuy et al., 2022; Poochada et al., 2024). Various vegetables commonly used for seasoning and garnishing in Thai cuisine, such as peppermint, yard-long beans, Chinese cabbage, cabbage, celery, cilantro, and cucumber, are frequently found to be contaminated. Thus, the location and type of markets are crucial factors influencing the transmission of infectious pathogens (IPs).

Nevertheless, limited information is available on the prevalence and distribution of these parasites in fresh vegetables in Bangkok and its surroundings, Thailand. Therefore, this study aimed to investigate the prevalence and distribution of intestinal parasites in fresh vegetables in Bangkok and its surrounding areas.

## 2 Materials and methods

### 2.1 Study area

This study was a cross-sectional survey carried out from November 1, 2022, to December 31, 2022, in the Bangkok metropolis and its surrounding areas (Nonthaburi, Pathum Thani, Nakhon Pathom, Samut Prakan, and Samut Sakhon), located in the central region of Thailand and situated at 13.7365° latitude and 100.5417° longitude. The region is characterized as a tropical rainforest zone with an annual average temperature of 29–37 °C (Climatological Center, 2024). This densely populated urban and suburban area in central Thailand has a population of approximately 5.5 million people (Department of Health, Ministry of Public Health, 2024), making it representative of large urban centers where health-related research can impact significant portions of the population. Furthermore, Bangkok and its surrounding areas serve as a critical economic and industrial hub, with substantial fresh vegetable imports from nearby agricultural regions, reflecting a complex supply chain vulnerable to contamination and cross-contamination.

**Fig. 1:**
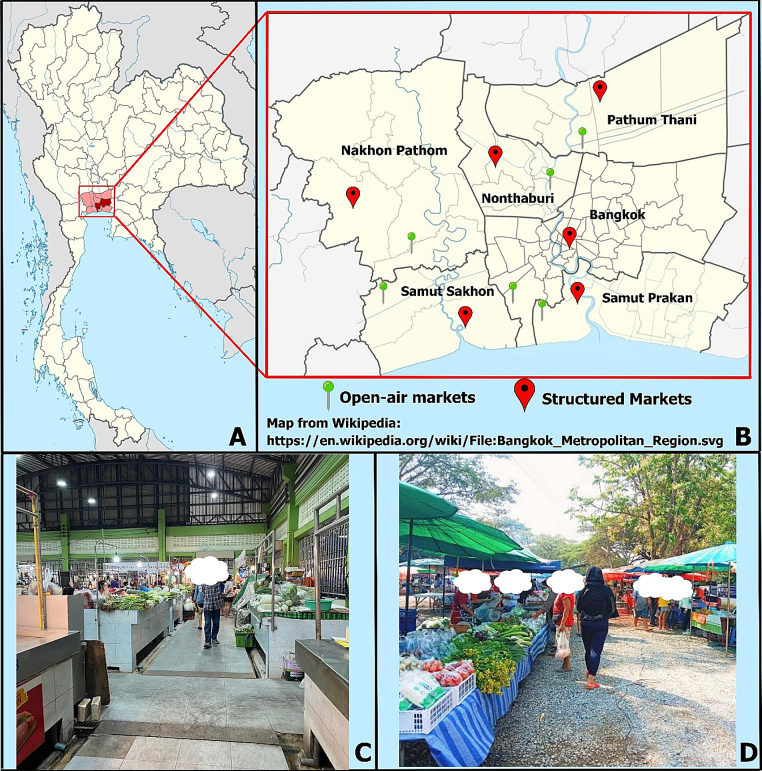
A: Bangkok and its surroundings, located in the central region of Thailand, B: open-air markets and structured markets located in Bangkok, Nonthaburi, Pathum Thani, Nakhon Pathom, Samut Prakan, and Samut Sakhon provinces, C: structured market, and D: open-air market.

The finite population proportion formula (Wayne, 1995) was applied to calculate the number of markets required for this study, using an estimated prevalence of IP (p) of 36.0 % in vegetables (Krainara et al., 2023). With a total of 55 markets in Bangkok and its surrounding areas (N) (Department of Internal Trade of Thailand, Ministry of Commerce, 2018) and a 95 % confidence level (CI) (z = 1.96), the minimum required sample size was calculated to be four markets. Ultimately, twelve markets were selected: six open-air markets in rural areas and six structured markets in urban areas across each province, chosen through simple random sampling, resulting in a total of 12 markets for the study. Fresh vegetable samples were collected from all 12 markets (Fig. 1).

A structured market was defined as a permanent market with a roof covering all areas, in which merchandise stalls are enclosed, constructed with permanent materials, smooth, sloped, and easy to clean. The panel area should be at least 2 square meters with a height of not less than 60 cm from the floor. Most sellers in structured markets source their vegetables from wholesale markets (Bureau of Food and Water Sanitation, Department of Health, Ministry of Public Health, 2008) (Fig. 1). Conversely, an open-air market was defined as having a height of less than 60 cm from the floor and lacking the structural features of a structured market.

Data on vegetable sourcing and hygiene practices across cultivation, harvesting, transportation, and sales stages were collected through structured interviews. In structured markets, most vendors sourced vegetables from farms in other provinces, while in open-air markets, most vendors cultivated vegetables on their own farms and sold them directly.

### 2.2 Sample collection

**Fig. 2:**
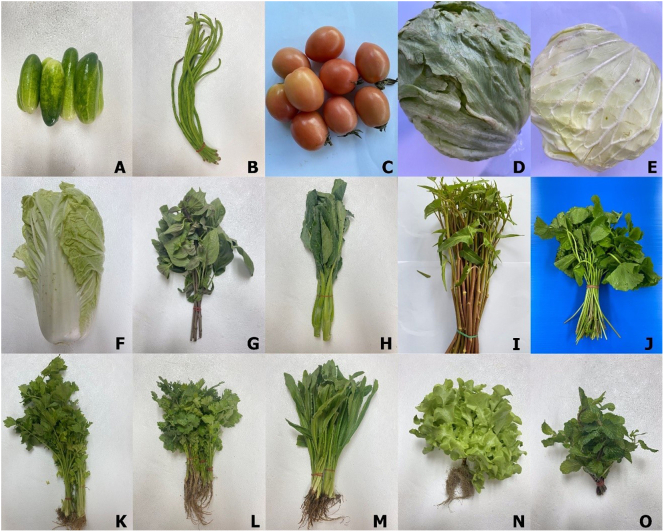
Images of fresh vegetable samples: A: cucumber, B: yard-long bean, C: tomato, D: iceberg lettuce, E: white cabbage, F: Chinese cabbage, G: Thai basil, H: Chinese kale, I: aquatic morning glory or water spinach, J: Indian pennywort, K: celery, L: coriander, M: culantro, N: lettuce, and O: peppermint.

The study included a total of 1800 fresh vegetable samples, representing 15 types of vegetables commonly consumed raw. Samples were randomly purchased, with 10 samples collected per vegetable type. The vegetable types included cucumber (*Cucumis sativus*), yard-long bean (*Vigna unguiculata* subsp. *sesquipedalis*), tomato (*Solanum lycopersicum*), iceberg lettuce (*Lactuca sativa* var. *capitata*), white cabbage (*Brassica oleracea*), Chinese cabbage (*Brassica rapa* subsp. *pekinensis*), Thai basil (*Ocimum basilicum*), Chinese kale (*Brassica oleracea* var. *alboglabra*), aquatic morning glory or water spinach (*Ipomoea aquatica*), Indian pennywort (*Centella asiatica*), celery (*Apium graveolens*), coriander (*Coriandrum sativum*), culantro (*Eryngium foetidum*), lettuce (*Lactuca sativa*), and peppermint (*Mentha x piperita*) (Fig. 2). Samples were meticulously collected in labeled, hygienic plastic bags and promptly transported to the parasitology laboratories at the Chulalongkorn University Faculty of Medicine and the Khon Kaen University Faculty of Public Health for parasitic examination.

### 2.3 Detection of IPs

The procedure employed for sample preparation and washing was adapted from Bekele and Shumbej (2019). After removing the root portions, fresh vegetable samples weighing 200 g were washed with a solution containing 1000 ml of 1 % Sodium Dodecyl Sulfate (SDS) and 0.1 % Tween 20® (Bio Basic Inc., Ontario, Canada) and shaken at 1300 cycles/min for 30 min to facilitate the separation of parasites from the vegetables. The washing water was then filtered through white cotton to remove vegetable debris. The filtrate was collected and allowed to settle overnight for sedimentation. The supernatant was carefully decanted, and any residual washing water was removed, transferring the sediment into 15 ml conical tubes. The sediment was centrifuged at 3000 ×*g* for 5 min. After centrifugation, the supernatant was gently aspirated without disturbing the sediment. The sediment was subsequently examined by two independent parasitologists under a microscope using a simple direct smear with 1 % iodine solution and 10× and 40× objectives (Garcia, 2007). All samples were stained with Lugol's iodine to detect IPs (El Said Said, 2012). The examination results were categorized into two groups: mono-contamination, where a single species of IP was detected, and poly-contamination, where two or more species of IPs were identified.

The morphology of each IP used for identification is as follows:

1. *Strongyloides* spp.: The rhabditiform larva is characterized by a rhabditiform esophagus and a prominent genital primordium, while the filariform larva possesses a filariform esophagus (long and smooth) and a notched tail (Beaver et al., 1984; Zeibig, 2012).
2. Hookworm larvae: The rhabditiform larva exhibits a rhabditiform esophagus and a long buccal cavity but lacks a genital primordium, whereas the filariform larva features a filariform esophagus, a pointed tail, and an enclosing sheath (Beaver et al., 1984; Zeibig, 2012).
3. *Ascaris lumbricoides*: The eggs are oval or round, measuring approximately 55–75 μm in length and 35–50 μm in width, and are encased in a thick shell (Beaver et al., 1984; Liu, 2018).
4. *Taenia* spp.: The eggs are spherical, surrounded by a thick striated wall, and approximately 40–48 μm in diameter, with a six-hooked (hexacanth) larva as the infective stage (Liu, 2018; Parija and Ponnambath, 2013).
5. *Blastocystis* sp.: Morphology varies depending on the life cycle stage. The vacuolar form, most frequently detected in this study, is spherical to oval with a large central vacuole occupying most of the cell's volume. The peripheral cytoplasm contains up to four nuclei, mitochondria-like organelles, and other inclusions (Liu, 2018; Tan, 2008).
6. *Giardia* spp.: The cysts are oval, measuring approximately 8–17 μm in length and 6–10 μm in width. They contain one to four nuclei, axonemes, and median bodies (Liu, 2018; Zeibig, 2012).
7. *Entamoeba coli*: The cysts are spherical to slightly oval, approximately 10–35 μm in diameter, and contain one to eight nuclei with an eccentric karyosome (Beaver et al., 1984; Zeibig, 2012).
8. *Entamoeba histolytica*: The cysts are typically spherical or oval, approximately 10–20 μm in diameter, and contain one to four nuclei with a centric karyosome (Beaver et al., 1984; Zeibig, 2012). However, *E. histolytica* similarities between *E. moshkovskii* and *E. dispar* make them difficult to identify.

### 2.4 Statistical analysis

Statistical analysis was performed using the STATA package version 18 (College Station, Texas: StataCorp LLC) under the license of Khon Kaen University. Descriptive statistics, including number of samples with IP contamination and percentage, were used to describe the qualitative data on intestinal parasitic infection. The independent variables in this study included the type of market, the category of fresh vegetables, and the province. The dependent variable was the presence of IP contamination in fresh vegetables, classified as either detected or not detected. Simple logistic regression was used to compare the prevalence of IP infection between different types of markets, allowing the assessment of the odds of infection occurrence in one market type relative to another. A *P*-value of less than 0.05 was considered statistically significant.

## 3 Results

A total of 1800 fresh vegetable specimens were included in this study, comprising 900 (50.0 %) sourced from six open-air markets in rural areas and 900 (50.0 %) from six structured markets in urban areas. The overall prevalence of IP contamination was 21.17 % (95 % Confidence Interval (95 % CI): 19.30–23.12), with mono-contamination accounting for 16.56 % and poly-contamination for 4.61 %. The prevalence of IP contamination was higher in open-air markets in rural areas (23.89 %, 95 % CI: 21.13–26.81) compared to structured markets in urban areas (18.44 %, 95 % CI: 15.96–21.13) (Table 1). Among the provinces, the highest contamination rate was observed in Bangkok (31.67 %, 95 % CI: 26.44–37.25), while the lowest was recorded in Nonthaburi (13.67 %, 95 % CI: 9.99–18.08) (Table 1).

**Table 1:** Prevalence of intestinal parasitic infections in 15 vegetables classified by market location.

Province	No. samples	Types of intestinal parasitic contaminations (%)		Total no. samples contaminated (%)	95 %CI
		No. samples with mono-contamination (%)	No. samples with poly-contamination (%)		
Nonthaburi					
Urban	150	9 (6.0)	6 (4.0)	15 (10.0)	5.70 to 15.96
Rural	150	20 (13.33)	6 (4.0)	26 (17.33)	11.65 to 24.36
Total	300	29 (9.67)	12 (4.0)	41 (13.67)	9.99 to 18.08
Samut Prakan					
Urban	150	18 (12.0)	1 (0.67)	19 (12.67)	7.80 to 19.07
Rural	150	20 (13.33)	3 (2.0)	23 (15.33)	9.97 to 22.11
Total	300	38 (12.67)	4 (1.33)	42 (14.0)	10.28 to 18.45
Nakhon Pathom					
Urban	150	27 (18.0)	6 (4.0)	33 (22.0)	15.65 to 29.48
Rural	150	20 (13.33)	7 (4.67)	27 (17.99)	12.21 to 25.10
Total	300	47 (15.67)	13 (4.33)	60 (20.0)	15.62 to 24.98
Pathum Thani					
Urban	150	22 (14.67)	7 (4.67)	29 (19.33)	13.35 to 26.57
Rural	150	37 (24.67)	5 (3.33)	42 (28.0)	20.98 to 35.91
Total	300	59 (19.67)	12 (4.0)	71 (23.67)	18.97 to 28.89
Samut Sakhon					
Urban	150	22 (14.67)	8 (5.33)	30 (20.0)	13.92 to 27.30
Rural	150	31 (20.67)	11 (7.33)	42 (28.0)	20.98 to 35.91
Total	300	53 (17.67)	19 (6.33)	72 (24.0)	19.28 to 29.24
Bangkok					
Urban	150	29 (19.33)	11 (7.33)	40 (26.67)	19.78 to 34.49
Rural	150	43 (28.67)	12 (8.0)	55 (36.67)	28.96 to 44.91
Total	300	72 (24.0)	23 (7.67)	95 (31.67)	26.44 to 37.25
Overall					
Urban	900	127 (14.11)	39 (4.33)	166 (18.44)	15.96 to 21.13
Rural	900	171 (19.0)	44 (4.89)	215 (23.89)	21.13 to 26.81
Total	1800	298 (16.56)	83 (4.61)	381 (21.17)	19.30 to 23.12

**Fig. 3:**
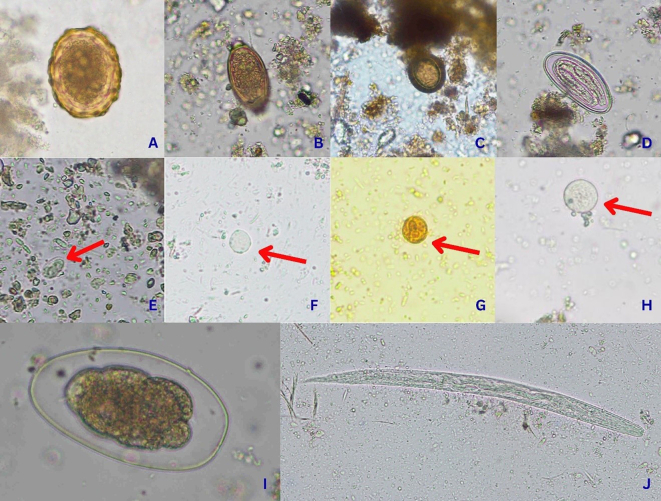
Images of intestinal parasites found in this study. *A: Ascaris lumbricoides*, B: *Trichuris* spp., C: Taeniidae spp., D: Oxyuridae eggs, E: *Giardia* spp., F: *Blastocystis* sp., G: *Entamoeba* spp., H: *Entamoeba coli*, I: Hookworm larvaes, and J: *Strongyloides* spp. (rhabditiform larvae).

The IPs detected overall fresh vegetable samples included hookworm larvae (13.06 %), *Strongyloides* spp. (rhabditiform larvae) (6.61 %), *Ascaris lumbricoides* (2.50 %), Taeniidae spp. (1.33 %), *Trichuris* spp. (0.72 %), Oxyuridae eggs (0.22 %), *Blastocystis* sp. (1.56 %), *Giardia* spp. (0.28 %), *Entamoeba coli* (0.17 %), and *Entamoeba* spp. (0.11 %) (Table 2). Images of the IPs identified in this study are shown in Fig. 3.

**Table 2:** Distribution of intestinal parasites in 15 fresh vegetables collected from markets in Bangkok and its surroundings, Thailand, from November to December 2022.

Vegetables	Number of samples	Intestinal parasitic contaminations										No. a parasitic contamination (%)
		No. *E.* spp. (%)	No. *Ec* (%)	No. *Gi* (%)	No. *Bl* (%)	No. *Tae* (%)	No. *Oxy* (%)	No. *Al* (%)	No. *Tr* (%)	No. *Str* (%)	No. Hw (%)	
Tomato	120	0 (0.0)	0 (0.0)	0 (0.0)	10 (8.33)	0 (0.0)	0 (0.0)	0 (0.0)	0 (0.0)	0 (0.0)	0 (0.0)	10 (8.33)
Cucumber	120	0 (0.0)	0 (0.0)	0 (0.0)	5 (4.17)	0 (0.0)	0 (0.0)	0 (0.0)	0 (0.0)	0 (0.0)	0 (0.0)	5 (4.17)
Yard long bean	120	0 (0.0)	0 (0.0)	0 (0.0)	0 (0.0)	2 (1.67)	0 (0.0)	0 (0.0)	0 (0.0)	1 (0.83)	0 (0.0)	3 (2.50)
Cabbage	120	0 (0.0)	0 (0.0)	0 (0.0)	7 (5.83)	0 (0.0)	0 (0.0)	0 (0.0)	0 (0.0)	0 (0.0)	2 (1.67)	9 (7.50)
Aquatic morning glory	120	0 (0.0)	0 (0.0)	0 (0.0)	0 (0.0)	0 (0.0)	0 (0.0)	3 (2.50)	0 (0.0)	2 (1.67)	0 (0.0)	5 (4.17)
Chinese kale	120	0 (0.0)	1 (0.83)	0 (0.0)	0 (0.0)	1 (0.83)	0 (0.0)	1 (0.83)	0 (0.0)	0 (0.0)	0 (0.0)	3 (2.50)
Lettuce	120	0 (0.0)	0 (0.0)	0 (0.0)	0 (0.0)	1 (0.83)	0 (0.0)	2 (1.67)	0 (0.0)	7 (5.83)	4 (3.33)	13 (10.83)*
Iceberg lettuce	120	2 (1.67)	0 (0.0)	2 (1.67)	0 (0.0)	0 (0.0)	0 (0.0)	0 (0.0)	0 (0.0)	0 (0.0)	0 (0.0)	4 (3.33)
Chinese cabbage	120	0 (0.0)	1 (0.83)	0 (0.0)	0 (0.0)	2 (1.67)	1 (0.83)	0 (0.0)	6 (5.0)	0 (0.0)	6 (5.0)	14 (11.67)*
Thai basil	120	0 (0.0)	0 (0.0)	0 (0.0)	0 (0.0)	1 (0.83)	0 (0.0)	2 (1.67)	0 (0.0)	0 (0.0)	87 (72.5)	87 (72.50)*
*Centella asiatica*	120	0 (0.0)	0 (0.0)	0 (0.0)	0 (0.0)	4 (3.33)	0 (0.0)	8 (6.67)	0 (0.0)	24 (20.0)	25 (20.83)	49 (40.83)*
Coriander	120	0 (0.0)	1 (0.83)	3 (2.50)	0 (0.0)	9 (7.5)	0 (0.0)	10 (8.33)	1 (0.83)	26 (21.67)	10 (8.33)	41 (34.17)*
Celery	120	0 (0.0)	0 (0.0)	0 (0.0)	2 (1.67)	1 (0.83)	0 (0.0)	6 (5.0)	1 (0.83)	2 (1.67)	8 (6.67)	18 (15.00)*
Culantro	120	0 (0.0)	0 (0.0)	0 (0.0)	4 (3.33)	3 (2.50)	1 (0.83)	5 (4.17)	1 (0.83)	17 (14.17)	2 (1.67)	27 (22.50)*
Peppermint	120	0 (0.0)	0 (0.0)	0 (0.0)	0 (0.0)	0 (0.0)	2 (1.67)	8 (6.67)	4 (3.33)	40 (33.33)	91 (75.83)	95 (79.17)*
Total	1800	2 (0.11)	3 (0.17)	5 (0.28)	28 (1.56)	24 (1.33)	4 (0.22)	45 (2.50)	13 (0.72)	119 (6.61)	235 (13.06)	381 (21.17)*

Remark: *E*. spp. = *Entamoeba* spp., *Ec* = *E. coli*, *Gi* = *Giardia* spp., *Bl* = *Blastocystis* sp., *Tae* = Taeniidae spp., *Oxy* = Oxyuridae eggs, *Al* = *A. lumbricoides*, *Tr* = *Trichuris* spp., *Str* = *Strongyloides* spp. (rhabditiform larvae), Hw = Hookworm larvae. *Some fresh vegetable samples were found to be co-contamination.

In this study, the three fresh vegetables with the highest prevalence of IP contamination were peppermint (79.17 %), Thai basil (72.50 %), and *Centella asiatica* (40.83 %). Peppermint was exclusively contaminated with nematode helminths, including hookworm larvae (75.83 %), *Strongyloides* spp. (rhabditiform larvae) (33.33 %), *A. lumbricoides* (6.67 %), *Trichuris* spp. (3.33 %), and Oxyuridae eggs (1.67 %). Thai basil was contaminated with hookworm larvae (72.50 %), *A. lumbricoides* (1.67 %), and Taeniidae spp. (0.83 %). *Centella asiatica* was contaminated with hookworm larvae (20.83 %), *Strongyloides* spp. (rhabditiform larvae) (20.0 %), *A. lumbricoides* (6.67 %), and Taeniidae spp. (3.33 %) (Table 2). Interestingly, iceberg lettuce was found to be contaminated with pathogenic protozoa, including *Entamoeba* spp. (1.67 %) and *Giardia* spp. (1.67 %). Additionally, tomatoes and cucumbers were exclusively contaminated with *Blastocystis* sp. at rates of 8.33 % and 4.17 %, respectively (Table 2).

For fresh vegetables in open-air markets located in rural areas, the highest prevalence rates were observed for hookworm larvae (14.0 %), followed by *Strongyloides* spp. (rhabditiform larvae) (5.89 %) and *A. lumbricoides* (3.11 %) (Table 3). For fresh vegetables in structured markets in urban areas, the highest prevalence rate was also hookworm larvae (12.11 %), followed by *Strongyloides* spp. (rhabditiform larvae) (7.33 %), *A. lumbricoides* (1.89 %), and Taeniidae spp. (0.78 %). Notably, samples from structured markets were the only ones found to contain *Entamoeba* spp. (0.22 %) (Table 3).

**Table 3:** Comparison of the rate of intestinal parasitic contamination in fresh vegetables from structured markets in urban areas and open-air markets in rural areas, classified by parasite, from November to December 2022.

Parasite	No. pos (*N* = 900 per area)	OR	95 %CI	P-value
***Entamoeba* spp.**				
urban	2 (0.22)	1		
rural	0 (0.0)	omitted		
***E. coli***				
urban	0 (0.0)	1		
rural	3 (0.33)	omitted		
***Giardia* spp.**				
urban	2 (0.22)	1		0.653
rural	3 (0.33)	1.5	0.25 to 9.01	
***Blastocystis* sp.**				
urban	3 (0.33)	1		<0.001
rural	25 (2.78)	8.54	2.57 to 28.40	
**Taeniidae spp.**				
urban	7 (0.78)	1		0.036
rural	17 (1.89)	2.46	1.01 to 5.95	
**Oxyuridae eggs**				
urban	0 (0.0)	1		
rural	4 (0.44)	omitted		
***A. lumbricoides***				
urban	17 (1.89)	1		0.095
rural	28 (3.11)	1.66	0.91 to 3.07	
***Trichuris* spp.**				
urban	3 (0.33)	1		0.045
rural	10 (1.11)	3.36	0.92 to 12.25	
***Strongyloides* spp. (rhabditiform larvae)**				
urban	66 (7.33)	1		0.217
rural	53 (5.89)	0.79	0.54 to 1.15	
**Hookworm larvae**				
urban	109 (12.11)	1		0.234
rural	126 (14.00)	1.18	0.90 to 1.56	

Open-air markets in rural areas were 8.54 times more likely to have contamination with *Blastocystis* sp. compared to structured markets in urban areas (Odds Ratio [OR]: 8.54, 95 % CI: 2.57–28.40, *P* < 0.001). Additionally, fresh vegetables from open-air markets were 2.46 times more likely to be contaminated with Taeniidae spp. than those from structured markets (OR: 2.46, 95 % CI: 1.01–5.95, *P* = 0.036) (Table 3).

Five open-air markets located in rural areas (Bangkok, Nonthaburi, Samut Prakan, Pathum Thani, and Samut Sakhon) exhibited a higher prevalence of intestinal parasitic contamination compared to structured markets, whereas the structured markets in Nakhon Pathom had a higher prevalence rate than the open-air markets. However, the difference in prevalence rates between open-air markets and structured markets in Bangkok and its surrounding areas was not statistically significant (Table 4). Interestingly, fresh vegetables in open-air markets were 1.40 times more likely to be contaminated with IPs compared to those in structured markets (OR: 1.40, 95 % CI: 1.10–1.74, *P* = 0.005) (Table 4).

**Table 4:** Comparison of the levels of intestinal parasitic contamination in fresh vegetables from structured markets in urban areas and open-air markets in rural areas in Bangkok and its surrounding areas, from November to December 2022.

Province	No. samples	Number of samples contaminated (%)	OR	95 %CI	P-value
**Nonthaburi**					
Urban	150	15 (10.0)	1		0.064
Rural	150	26 (17.33)	1.88	0.95 to 3.73	
**Samut Prakan**					
Urban	150	19 (12.67)	1		0.506
Rural	150	23 (15.33)	1.25	0.64 to 2.40	
**Nakhon Pathom**					
Urban	150	33 (22.0)	1		0.386
Rural	150	27 (17.99)	0.77	0.44 to 1.37	
**Pathum Thani**					
Urban	150	29 (19.33)	1		0.063
Rural	150	42 (28.0)	1.88	0.95 to 3.73	
**Samut Sakhon**					
Urban	150	30 (20.0)	1		0.106
Rural	150	42 (28.0)	1.55	0.91 to 2.65	
**Bangkok**					
Urban	150	40 (26.67)	1		0.062
Rural	150	55 (36.67)	1.59	0.97 to 2.60	
**Overall**					
Urban	900	167 (18.56)	1		0.005
Rural	900	214 (23.78)	1.40	1.10 to 1.74	

## 4 Discussion

This study aimed to determine the prevalence of IPs in fresh vegetables sold in Bangkok and on surrounding areas. The results revealed a high overall prevalence of 21.17 %, though this is lower than those reported in previous studies conducted in Asia, including Nakhon Si Thammarat, southern Thailand (35.10 %) (Punsawad et al., 2019), Manila, Philippines (45.0 %) (Sia Su et al., 2012), and Hanoi, Vietnam (26.0 %) (Uga et al., 2009). Furthermore, the prevalence observed in this study is also lower than findings from Libya (58.0 %) (Abougrain et al., 2010), Brazil (50.9 %) (Luz et al., 2017), Ethiopia (42.6–54.4 %) (Bekele and Shumbej, 2019; Bekele et al., 2017), and Egypt (29.6–60.70 %) (Eraky et al., 2014; Yahia et al., 2023). This difference may be attributed to the smaller sample sizes used in those studies, which analyzed fewer than 600 vegetable samples. However, the prevalence of IPs in this study was higher than reported in studies conducted in Sudan (13.5 %) (Mohamed et al., 2016), Turkey (5.9 %) (Kozan et al., 2005), northern Iran (14.9 %) (Rostami et al., 2016), and Egypt (19.4 %) (Hassan et al., 2012). This higher prevalence may be due to various factors, including poor sanitation and hygiene practices during the cultivation, harvesting, transportation, and sale (Altwaim et al., 2023; Bekele and Shumbej, 2019; Punsawad et al., 2019).

The three fresh vegetables with the highest IP contamination rates were peppermint (75.83 %), Thai basil (72.50 %), and *Centella asiatica* (20.83 %). This finding differs from the studies by Altwaim et al. (2023), Yahia et al. (2023), and Punsawad et al. (2019), which reported the highest prevalence in coriander, parsley, and celery, respectively. The increased prevalence observed in peppermint, Thai basil, and *Centella asiatica* could be attributed to the coarse texture of their leaf surfaces, which may facilitate the adherence of parasites (Punsawad et al., 2019). Fresh vegetables are commonly used to season and garnish Thai dishes in various areas of Thailand including the central, northeastern, and southern regions.

Soil-transmitted helminths, including hookworm larvae, *Strongyloides* spp. (rhabditiform larvae), *Ascaris lumbricoides*, and *Trichuris* spp., were highly prevalent in vegetables from both open-air and structured markets, particularly in shrubs such as peppermint, Thai basil, and *Centella asiatica*. These findings align with previous reports from Thailand (Pednog and Toonsakool, 2020; Punsawad et al., 2019). The presence of pathogenic parasites such as *Blastocystis* sp., Taeniidae spp., Oxyuridae eggs, *Giardia* spp., and *Entamoeba* spp., in Bangkok and surrounding areas underscores the importance of implementing effective sanitary management practices. This study determined that helminths exhibit higher levels of contamination compared to protozoa. This can be attributed to the thicker protective shells of helminths, which enhance their resistance to environmental conditions and increase their survival rates relative to protozoa (Jansen et al., 2021; Larsen and Roepstorff, 1999). Similar findings have been reported in previous studies, highlighting the persistent issue of protozoan contamination in fresh vegetables (Bekele and Shumbej, 2019; Eraky et al., 2014; Mohamed et al., 2016; Sia Su et al., 2012). This contamination may result from agricultural practices in areas supplying vegetables to Bangkok and surrounding areas, where human or animal feces are used as fertilizer or contaminated water is employed for irrigation (Punsawad et al., 2019). The findings revealed that open-air markets in rural areas had a higher prevalence of IPs compared to structured markets in urban areas, which was similar to previous studies (Duedu et al., 2014; Niamnuy et al., 2022; Poochada et al., 2024). This disparity may result from unsuitable agricultural practices, inadequate post-harvest handling, and the use of water to moisten vegetables during sales (Altwaim et al., 2023; Okyay et al., 2004). These results suggest that quality control measures, including hygienic handling and washing practices in structured markets, may be more effective at reducing contamination risks (Punsawad et al., 2019) and underscore the need for enhanced hygiene practices and quality control measures in open-air markets in rural areas (Duedu et al., 2014; Falcone et al., 2023; Punsawad et al., 2019).

This study highlighted a high prevalence of IPs in fresh vegetables in the central region of Thailand, indicating their potential to transmit IPs to humans. Therefore, vegetables should be thoroughly washed before consumption. Previous studies have indicated that standard washing methods can significantly reduce the risk of parasitic contamination in fresh vegetables (Avcioğlu et al., 2011; Fallah et al., 2016). These methods include the use of disinfecting agents such as acetic acid (5 %) and potassium permanganate (24 mg/L) for 15 and 30 min, respectively (Etewa et al., 2017). Consequently, health institutions must disseminate guidelines on proper hygiene protocols within communities to reduce contamination and transmission risks. However, this study has certain limitations. Several factors that may affect parasite contamination in vegetables—such as planting locations, water sources, harvesting seasons, storage, transportation to markets, and preparation methods, including washing—were not controlled in this study. These limitations highlight the importance of future research to examine the impact of irrigation water quality, harvesting practices, and post-harvest handling on vegetable contamination. This addition will help provide a more comprehensive understanding of the factors contributing to parasitic contamination. Additionally, while morphological examination under microscopy is a well-established method for identifying protozoa and nematodes, it requires the expertise of a parasitologist to differentiate species accurately. To address these challenges, biomolecular approaches such as polymerase chain reaction (PCR) and real-time PCR should be employed to detect specific species, particularly *E. histolytica*, hookworm larvaes, and *S. stercoralis*. These techniques are crucial for distinguishing *E. histolytica* from other *Entamoeba* species, as well as for differentiating hookworm larvaes and *S. stercoralis* from other morphologically similar roundworms.

## 5 Conclusions

The findings of this study highlight that the high contamination of fresh vegetables with IPs, particularly helminths, in Bangkok and the surrounding areas of the central region of Thailand poses a significant risk of transmission to consumers. Vegetables from open-air markets in rural areas showed higher levels of contamination than those from organized markets in urban areas. To mitigate this risk, it is crucial to raise consumer awareness about preventive measures, including thoroughly washing vegetables with acetic acid (5 %) and potassium permanganate (24 mg/L) for 15 and 30 min, respectively, and properly cooking them by boiling or steaming before consumption. Additionally, implementing comprehensive health education programs is essential to promote hygiene practices among sellers and farmers. These practices include wearing gloves and washing hands after handling vegetables to reduce the likelihood of contamination and transmission.

## Declaration of cmpeting interest

The authors declare that they have no known competing financial interests or personal relationships that could have appeared to influence the work reported in this paper.

## Declaration of generative AI in scientific writing

During the preparation of this work the authors used ChatGPT in order to rewrite sentences and improve grammar. After using this tool, the authors reviewed and edited the content as needed and took full responsibility for the content of the publication.

## CRediT authorship contribution statement

**Pokkamol Laoraksawong:** Writing – review & editing, Writing – original draft, Visualization, Validation, Resources, Project administration, Methodology, Investigation, Formal analysis, Conceptualization. **Uthaitip Bunkasem:** Resources, Project administration, Methodology, Investigation, Data curation. **Anunya Pradidthaprecha:** Writing – review & editing, Writing – original draft, Visualization, Software, Project administration, Methodology, Formal analysis, Conceptualization.

## Data Availability

The data that support the findings of this study are available from the corresponding author upon reasonable request.
